# Association between Cardiovascular Risk Assessment by SCORE2 and Diverticulosis: A Cross-Sectional Analysis

**DOI:** 10.3390/jpm14080862

**Published:** 2024-08-14

**Authors:** Andreas Völkerer, Sarah Wernly, Georg Semmler, Maria Flamm, Konrad Radzikowski, Leonora Datz, Nikolaus Götz, Hannah Hofer, Elmar Aigner, Christian Datz, Bernhard Wernly

**Affiliations:** 1 Department of Internal Medicine, General Hospital Oberndorf, Teaching Hospital of the Paracelsus Medical University, 5020 Salzburg, Austria; sarah@wernly.net (S.W.); k.radzikowski@salk.at (K.R.); l.datz@kh-oberndorf.at (L.D.); hannah.hofer@bbsalz.at (H.H.); c.datz@kh-oberndorf.at (C.D.); bernhard@wernly.net (B.W.); 2 Division of Gastroenterology and Hepatology, Department of Medicine III, Medical University of Vienna, 1090 Vienna, Austria; georg.semmler@meduniwien.ac.at; 3 Institute of General Practice, Family Medicine and Preventive Medicine, Center for Public Health and Healthcare Research Paracelsus Medical University, 5020 Salzburg, Austria; maria.flamm@pmu.ac.at; 4 Department of Internal Medicine, General Hospital St. Vinzenz Zams, 6511 Zams, Austria; nikolaus.goetz@krankenhaus-zams.at; 5 Clinic I for Internal Medicine, University Hospital Salzburg, Paracelsus Medical University, 5020 Salzburg, Austria; e.aigner@salk.at

**Keywords:** diverticulosis, SCORE2, metabolic syndrome, cardiovascular risk

## Abstract

Background: The aim of this retrospective observational study was to examine the relationship between SCORE2 and the occurrence of colonic diverticula in a screening population without cardiovascular or gastrointestinal symptoms. SCORE2, recognized and supported by the European Society of Cardiology for cardiovascular risk assessment, served as the primary metric for the analysis in this investigation. Methods: We studied 3935 asymptomatic individuals undergoing screening colonoscopy. SCORE2 was calculated for each participant and categorized into three groups based on the following projected 10-year cardiovascular disease risk: SCORE2 0–4.9%, SCORE2 5–9.9%, and SCORE2 ≥ 10%. Logistic regression was used to assess the relationship between SCORE2 and diverticulosis. Results: SCORE2 was associated with the presence of diverticulosis (OR 1.09, 95%CI 1.07–1.10; *p* < 0.001) in univariable logistic regression, translating into an RR of 1.07 per unit increase. The association persisted after multivariable adjusting for metabolic syndrome (aOR 1.08; 95%CI 1.06–1.10; *p* < 0.001). Patients with high cardiovascular risk had higher rates of diverticulosis compared to those with lower risk: high risk (OR 2.00, 95%CI 1.71–2.33; *p* < 0.001); very high risk (OR 2.53, 95%CI 2.10–3.05; *p* < 0.001). This association remained after adjusting for metabolic syndrome: high risk (aOR 1.86, 95%CI 1.59–2.18; *p* < 0.001); very high risk (aOR 2.27, 95%CI 1.88–2.75; *p* < 0.001). Conclusions: A higher SCORE2 was found to be a suitable screening parameter for diverticular disease. This suggests a potential link between cardiovascular risk factors and colon diverticula development, warranting further research on whether optimizing cardiovascular risk factors could positively influence diverticular disease.

## 1. Introduction

This retrospective observational study primarily aimed to investigate the association between SCORE2 and the presence of colonic diverticula in an asymptomatic screening population. Cardiovascular diseases persist as the predominant cause of morbidity and mortality in Europe, with their risk factors substantially intersecting with those associated with diverticulosis. Given the potential reversibility of risk factors and unhealthy behaviors, there exists a substantial opportunity to mitigate the impact of these diseases [1]. The SCORE2 risk estimation chart is designed for individuals aged 40 to 69 and considers gender, age, smoking status, systolic blood pressure, and non-HDL cholesterol levels for calculation. SCORE2 is utilized to evaluate the 10-year risk of experiencing fatal and non-fatal cardiovascular events within the European population. Risk classifications are categorized based on age, with low-to-moderate risk defined as a SCORE2 < 2.5% for those under 50 and a SCORE2 < 5% for individuals aged 50–69. High risk is indicated by a SCORE2 ranging from 2.5 to 7.5% for those under 50 and 5 to 10% for those aged 50–69. Very high risk is defined as a SCORE2 > 7.5% for those under 50 and >10% for individuals aged 50–69 [2].

Colonic diverticulosis, characterized by the formation of diverticula in the intestinal wall, is a prevalent condition, particularly among the elderly. This condition often lacks symptoms [2,3], with approximately 10% of individuals under 40 and around 50–70% of those over 80 experiencing asymptomatic diverticulosis [4]. However, the incidence has risen to 50% in individuals aged over 60, with a notable increase observed in younger age groups [5].

Factors contributing to diverticulosis include genetics [6], age [7], and associations with cardiometabolic risks like hypertension [8], obesity [8], abdominal fat accumulation [9], and fatty liver disease [10,11]. Dietary patterns, such as alcohol consumption, are also linked to diverticula development [12]. Contrary to previous beliefs, recent data challenge the notion that a high-fiber diet protects against diverticulosis [13].

While the debate continues on as to whether asymptomatic diverticulosis should be considered a distinct disease [14,15], it is acknowledged that diverticular disease, when symptomatic, can be categorized into the following two types based on severity: symptomatic uncomplicated diverticular disease (SUDD) and symptomatic complicated disease, encompassing acute diverticulitis (with or without complications) or diverticular hemorrhage [16]. Though diverticulosis usually remains asymptomatic, approximately 10–25% of patients may eventually develop symptoms, including SUDD or, in severe cases, diverticulitis (4%), perforation, and bleeding [17,18,19]. Apart from ethnic background, age-related biological factors, and dietary influences, the formation of diverticula has also been associated with lifestyle elements [20], specifically factors related to cardiovascular and metabolic risks.

The prevention of diverticulosis has gained significance due to its increasing prevalence worldwide, as well as occasional but problematic complications [2]. In addition to the growing number of affected individuals, this also gives rise to a health system issue with non-negligible costs [21].

Therefore, we proposed the idea that there could be a distinct connection between SCORE2 and the occurrence of diverticulosis, taking into account the previously noted link between cardiovascular risk factors and those associated with diverticulosis. To address this scientific question, we utilized data from our single-center, epidemiological, retrospective, observational, non-interventional, and uncontrolled cohort study. This includes asymptomatic participants from the ‘Salzburger Kolon-Karzinom Prävention und Intervention’ (SAKKOPI) study. Conducted at the General Hospital Oberndorf near Salzburg, Austria, the primary focus of this study is on colorectal cancer screening, which is recommended starting at the age of 45 in Austria. Our findings are expected to offer additional understanding of the connection between cardiometabolic disease and diverticulosis. This should aid in recognizing potential approaches for preventive measures in individuals who are at risk of developing diverticulosis. Our research could also set a precedent for future basic science projects.

## 2. Materials and Methods

### 2.1. Subjects

**Figure 1:**
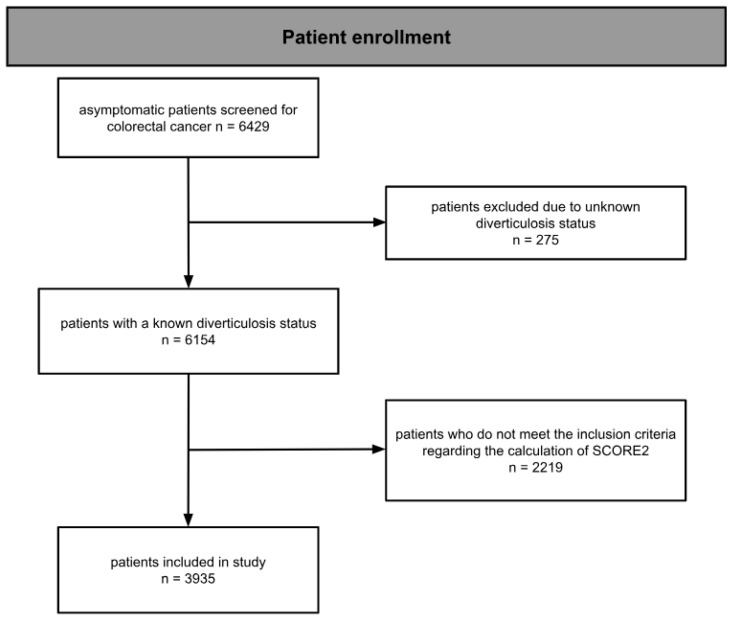
Flow diagram of patient enrollment.

The study is a single-center, epidemiological, retrospective, observational, non-interventional, and uncontrolled cohort study, conducted without blinding. It involves participants from the “Salzburger Kolon-Karzinom Prävention und Intervention” (SAKKOPI) study, which focuses on colorectal cancer screening, which is advised to start at age 45 in Austria. To date, 6429 subjects have undergone routine screening colonoscopies from one to three times, and have provided written informed consent for their data to be used for scientific research. The cohort, as also extensively described in previous studies [22], includes patients for whom the SCORE2 could be calculated and information regarding diverticulosis of the colon was available. Patients were excluded due to a previous history of cardiovascular issues (such as coronary artery disease, peripheral artery disease, transient ischemic attack, or stroke). Patients who did not meet the age criteria for the SCORE2 (40–69 years) were also excluded. This resulted in a patient population of 3935 individuals that was used for the analysis (Figure 1).

### 2.2. Patient Assessment for Risk Factors

In this study, patients underwent a comprehensive assessment over two consecutive days. The initial day involved the examination of vital signs, clinical evaluation, and laboratory tests upon admission to the hospital. To collect the necessary data, a detailed medical history was obtained through an interview (clinical history). The anamnesis and the clinical physical examination were documented in the medical computer program Patidok 2.0. Similarly, the digitalization of vital parameters was carried out after collection using certified blood pressure monitors and pulse oximeters. The ECG examination was directly imported from the certified device into the medical program and medically evaluated. Height and weight, as well as measurements of waist and hip circumference, were entered into the system by the qualified nursing staff, and the BMI was subsequently calculated automatically. The data needed for the study were then manually extracted from the digitized medical records by the research staff and transferred anonymously into a database (Microsoft Access). In a supplementary paper-based questionnaire with dichotomous questions, group questions, rating scales with stepped responses, as well as single- and multiple-choice options with additional selections, further required information (diet, physical activity, and social environment) was collected. Data were also collected using numerical values, particularly regarding eating habits and physical activity. Patients also provided information on their family and medical history through the questionnaire. In cases of uncertainties or missing information regarding the questionnaire or the clinical history, the study participant was contacted by telephone or mail. The blood samples taken were largely analyzed in the hospital’s internal laboratory. The laboratory diagnostics included routine laboratory parameters (complete blood count, electrolytes, liver function parameters, kidney function parameters, pancreatic enzymes, inflammation markers, lipids, and blood coagulation), parameters for iron and copper metabolism, the erythrocyte sedimentation rate, protein electrophoresis, and parameters for glucose metabolism (insulin levels, HbA1c, and oral glucose tolerance test). Each patient received a standardized abdominal ultrasound and a FibroScan with the Controlled Attenuation Parameter (CAP) and the elasticity median value to assess fibrotic and steatotic liver changes. Subsequently, colonoscopies were conducted on the following day. All of the examinations were carried out by specialists in internal medicine. Body mass index (BMI) was determined in accordance with the World Health Organization (WHO) guidelines, and arterial hypertension was classified following the ESC guideline on arterial hypertension management [23]. Smoking status was categorized as “ever smokers” or “active smokers” based on self-reported information, and the metabolic syndrome was defined per the IDF/AHA/NHLBI consensus [24].

### 2.3. Assessment of Cardiovascular Risk

The SCORE2 model is employed to predict the likelihood of experiencing fatal and non-fatal cardiovascular disease over a 10-year period in the European population [25]. This predictive tool is designed for individuals between the ages of 40 and 69 who have not previously encountered cardiovascular issues. The algorithm considers various risk factors, including gender, age, smoking habits, systolic blood pressure, as well as levels of total and high-density lipoprotein (HDL) cholesterol. Risk classifications are categorized based on age, with low-to-moderate risk defined as a SCORE2 < 2.5% for those under 50 and a SCORE2 < 5% for individuals aged 50–69. High risk is indicated by a SCORE2 ranging from 2.5 to 7.5% for those under 50 and 5 to 10% for those aged 50–69. Very high risk is defined as a SCORE2 > 7.5% for those under 50 and >10% for individuals aged 50–69. To implement the SCORE2 algorithm, the Stata code was obtained from the researchers conducting the study and executed accordingly.

### 2.4. Assessment of Diverticulosis

A colonoscopy was conducted in accordance with international guidelines, and all of the specified performance criteria were met [26]. The patients were classified into the categories of “no diverticulosis”, “left-sided diverticulosis”, “right-sided diverticulosis”, or “pandiverticulosis”, depending on the findings.

### 2.5. Statistical Analysis

In our study, we utilized Stata/BE 18.0 to perform a logistic regression analysis to assess the relationship between the presence of diverticulosis (dependent variable) and SCORE2, evaluated both as a continuous and categorical variable (low-to-moderate risk versus high and very high risk). This analysis yielded odds ratios (ORs) and adjusted odds ratios (aORs) with 95% confidence intervals (CIs). Clinically, ORs indicate the likelihood of diverticulosis associated with different SCORE2 values, with an OR above 1 suggesting a higher likelihood in the high-risk categories. In the context of our study, an odds ratio (OR) of 1.1 for the continuous SCORE2 variable indicated that, for each unit increase in the SCORE2, there was a 10% increase in the odds of having diverticulosis. This interpretation is based on the assumption that SCORE2 is a continuous variable, where each unit increase is meaningful in terms of risk assessment. The models were also adjusted for the presence of metabolic syndrome. Notably, we did not adjust for age and sex, as these factors are components of SCORE2, and their inclusion could have led to model overfitting. Further, we computed the relative risk (RR) using the ‘adjrr’ command. This advanced statistical function allowed us to adjust the RR for potential confounders, including the presence of metabolic syndrome, which is crucial for a precise assessment of the risk associated with different SCORE2 levels. The ‘adjrr’ command in Stata is particularly effective for deriving adjusted relative risk estimates from logistic regression models, especially pertinent when the outcome, in this case, diverticulosis, is not a rare event.

The calculation of RR provides an additional layer of insight by comparing the probability of developing diverticulosis among different SCORE2 categories, offering a more direct and clinically intuitive measure of risk. This method enhances our understanding of the relationship between SCORE2 levels and the risk of diverticulosis, beyond what is provided by odds ratios alone, and is especially useful in translating our findings into practical clinical and public health applications.

Continuous data are given as a median ± interquartile range (IQR) and compared using the Mann–Whitney U-test or mean ± standard deviation (SD) and compared using Student’s *t*-test accordingly. Categorical data are given as numbers (percentages) and compared using the chi-square test. All tests were two-sided, and a *p*-value of <0.05 was considered statistically significant.

## 3. Results

Out of the 3935 included asymptomatic patients who underwent endoscopic evaluation, diverticulosis was diagnosed in a total of 1326 (34%) patients, showing variations in severity and location. In total, 1512 (38%) patients had a low-to-moderate, 1651 (42%) had a high, and 772 (20%) had a very high 10-year risk of fatal and non-fatal cardiovascular disease (Table 1).

According to the given risk groupings, there is a significantly lower proportion of women in the high-risk (32%) and very-high-risk groups (20%). Furthermore, patients in the higher-risk categories are also increasingly older (53a vs. 57a vs. 63a; *p* < 0.001), exhibit significantly higher BMI values (25 vs. 27 vs. 28; *p* < 0.001), and more frequently meet the criteria for metabolic syndrome (62% vs. 82% vs. 93%; *p* < 0.001). While the number of active smokers was highest among those in the very-high-risk category (44%), the largest number of never smokers was found in the group with low-to-moderate risk (39%). 

**Figure 2:**
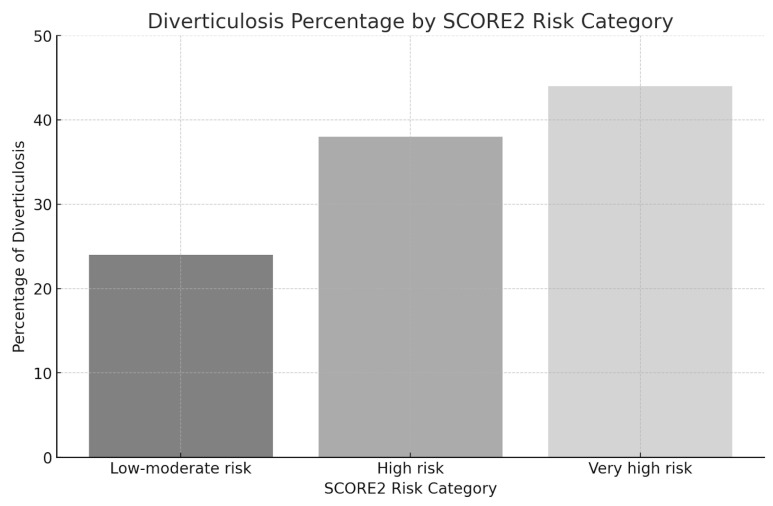
Percentage of patients with diverticulosis in the corresponding SCORE2 category.

The occurrences of diverticulosis were 24%, 38%, and 44% across the three SCORE2 categories (Table 2a,b) (Figure 2). As a continuous variable, SCORE2 was associated with the presence of diverticulosis (OR 1.09 95%CI 1.07–1.10; *p* < 0.001) in univariable multilevel logistic regression, translated into an RR of 1.07 (95%CI 1.05–1.08; *p* < 0.001). After multivariable adjustment for metabolic syndrome, a higher SCORE2 remained associated with higher odds for diverticulosis (aOR 1.08; 95%CI 1.06–1.10; *p* < 0.001).

Patients with high (42%) and very high risk (20%) for fatal and non-fatal cardiovascular disease over a 10-year period had significantly higher rates of colon diverticula compared to patients with low-to-intermediate risk (38%). 

In the initial analysis (Model-1), a higher likelihood of diverticulosis was observed with the high-risk (OR 2.00; 95% CI 1.71–2.33; *p* < 0.001) and very-high-risk groups (OR 2.53; 95% CI 2.10–3.05; *p* < 0.001). Even after accounting for metabolic syndrome (Model-2), an increased SCORE2 remained correlated with the presence of diverticulosis in both the high-risk group (aOR 1.86; 95% CI 1.59–2.18; *p* < 0.001) and the very-high-risk group (aOR 2.27; 95% CI 1.88–2.75; *p* < 0.001) (Table 3).

**Figure 3:**
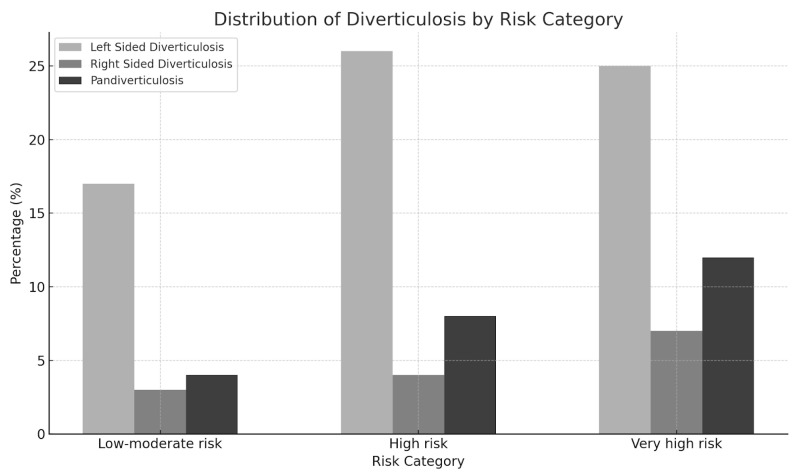
Distribution of left-sided diverticulosis, right-sided diverticulosis, and pandiverticulosis across the different SCORE2 risk categories.

The distribution of diverticula in the various segments of the colon (Figure 3) was not taken into account in this regard.

## 4. Discussion

In this cohort of 3935 asymptomatic patients who underwent endoscopic examination primarily for colorectal cancer screening, which is recommended to begin at age 45 in Austria, a significant increase in the presence of diverticula was observed with higher SCORE2 values. The SCORE2 index mainly reflects an elevated 10-year risk of fatal and non-fatal cardiovascular events. This association remained significant even after adjusting for metabolic syndrome.

Although many different cardiovascular risk scores are available and used in studies, it is important to consider their respective validation and limitations. As SCORE2 represents the newest and most advanced tool for predicting cardiovascular events, and can be easily applied in clinical practice, our investigation took a closer look at the association between SCORE2 and diverticulosis. 

Even though the data on this topic are scarce, Ukashi et al. presented a similar study that demonstrated an association between diverticulosis and cardiovascular risk using the ASCVD risk score and METs score during a treadmill exercise test [27]. Comparable results, in line with this trend, were obtained in a study by Tam et al., suggesting an association between diverticulitis and incident cardiovascular diseases, although the results did not reach statistical significance. Interestingly, the association between diverticulitis and the risk of cardiovascular diseases was more evident in participants without traditional risk factors for cardiovascular diseases [28]. A substantial prospective study, encompassing over 77,000 patients diagnosed with diverticular disease in Danish medical registries, uncovered a slightly elevated risk of arterial and venous thromboembolic events in individuals with diverticular disease. Nevertheless, these findings are limited by the reliance on diagnostic codes from medical and insurance registries, which hinders the ability to differentiate between diverticulitis, diverticular bleeding, and uncomplicated diverticulosis. Additionally, these studies failed to consider crucial variables for both diverticulosis and cardiovascular disease [29].

Current theories propose that the occurrence of diverticulitis and, to some extent, diverticulosis is linked to persistent inflammation in the intestines, which could contribute to a state of low-grade inflammation [30]. As chronic inflammation is associated with the development of cardiovascular disease, it raises the possibility of a causal relationship between diverticular disease and cardiovascular conditions [31]. Studies by Wang et al. indicate that the gut microbiota processes compounds associated with a proatherogenic state [32]. These changes have the potential to affect immune defenses, disturb the mucosal barrier, and initiate systemic inflammation. Consequently, they contribute to the emergence of chronic low-grade systemic inflammation and the formation of atherosclerotic plaques [33].

On the other hand, it has been hypothesized that the sites where the vasa recta pass through the lamina muscularis could be susceptible to the formation of diverticula. This could be attributed to alterations in the vessels and surrounding structures in this area [34]. In line with the positive correlation between diverticulosis and patient age [8,35], the aging process of the vessels, facilitated by traditional cardiovascular risk factors, leads to endothelial dysfunction, remodeling of the extracellular matrix, calcification, and overall increased vascular stiffness [36]. These changes in the blood vessels result in structural alterations and further weaken the already vulnerable areas that tend to bulge. However, it should be considered that both conditions share various classical risk factors, some of which are also included in SCORE2, such as age, arterial hypertension, obesity, hyperlipidemia, and gender. Therefore, different and distinct pathophysiological mechanisms may be responsible for each of them [37].

According to data from the Centers for Disease Control and Prevention, diverticulosis is evolving into a significant health issue in the USA, incurring substantial costs. Healthcare facilities in the United States documented approximately 1.07 million cases of diverticular disease in 2016, resulting in 207,150 hospitalizations (principal diagnosis), and total costs of just over USD 9.0 billion in 2018 [21]. These costs appear comparatively insignificant when compared to those caused by cardiovascular diseases (CVDs). The estimated costs associated with cardiovascular diseases (CVDs) amounted to around USD 251.4 billion in 2018–2019, doubling in the last 20 years [38]. However, this also provides a very cost-effective means for risk evaluation in diverticulosis patients, and vice versa. Calculating SCORE2 can be used, on the one hand, in diverticulosis patients to determine and subsequently optimize cardiovascular risk factors. On the other hand, in patients with cardiovascular baseline issues and a high SCORE2, it can further lead to gastroenterological assessment and optimization of risk and lifestyle factors. This might result in significant benefits both in terms of health economics and at an individual level in preventive medicine.

There are several limitations to this study. Firstly, the SCORE2 tool, a recent addition, could only be applied retrospectively to the SAKKOPI cohort, making this study inherently retrospective and post hoc. Furthermore, the cardiovascular risk was assessed only at the time of colonoscopy, rather than through a longitudinal design. This cross-sectional approach limits the ability to establish a temporal relationship between the observed cardiovascular risk factors and the occurrence of colorectal conditions. The cross-sectional design of our study only allows for a snapshot of the variables under investigation at a particular point in time. As a result, no causal relationships can be inferred, and the dynamics or changes over time remain unexplored. Given the cross-sectional design, there is a risk of confounding factors that may not be fully controlled. Additionally, selection bias and information bias could impact the validity of the results. Selecting a single center as the study site limits the generalizability of our findings to other geographical or population-based contexts. Local conditions and patient characteristics may be specific to the chosen center and, thus, may not be representative of the overall population. Since our study does not include basic science aspects, there is a lack of in-depth examination at the cellular or molecular level. This limitation restricts the understanding of underlying mechanisms and may complicate the interpretation of observed phenomena. Another limitation of the study is the low R^2^ value, which means that the model explains only a small portion of the variability of the dependent variable. This suggests that many other factors not included in the model may play a role. However, a low R^2^ value does not necessarily render the model unusable. The statistical significance confirms the relevance of the predictors. Additionally, the effect size, indicated by the odds ratio, points to a substantial and clinically significant effect. In complex phenomena like diverticulosis, even small explained variances contribute valuable insights to the overall understanding. Our results provide a valuable foundation for further studies that could incorporate additional factors. The low R^2^ value suggests that there are other relevant variables that future research should consider. By including more predictors or employing more complex models, the explanatory power could be enhanced. These subsequent studies could contribute to a more comprehensive understanding of the phenomenon under investigation and ultimately lead to more accurate predictive models.

However, we believe that this study possesses distinct merits. We were able to obtain detailed cardiometabolic data from a substantial group of asymptomatic individuals who underwent screening colonoscopy. Consequently, we could calculate the SCORE2 in a post hoc manner and, in our view, showcase in this study that SCORE2 not only predicts cardiovascular risk but also the risk of diverticulosis. The contemporary design of the study allows for a current and pertinent evaluation of the variables under investigation. Through timely data collection, the results can be more effectively applied to current conditions and developments in medical practice. The study stands out for its substantial number of patients, enhancing the robustness of the results. A high patient volume increases the statistical power of the study, enabling a more precise assessment of relationships and trends. A significant advantage of the study is that the data were collected through the daily clinical work of physicians. This ensures that the gathered data directly originate from clinical practice and not from an isolated database, thereby strengthening the external validity of the results. The fact that the data come directly from daily clinical practice imparts a high degree of practical relevance to the study. As a result, the findings reflect authentic situations, which is of great importance for the applicability of the insights in real clinical scenarios.

## 5. Conclusions

The results of this study, indicating a higher prevalence of diverticula in patients with increased cardiovascular risk, have significant implications for both public health and individual patient care. By elucidating the connection between cardiovascular risk and diverticula, this research contributes to a better understanding and prevention of risk factors for diverticulum formation and likely complications. Furthermore, it underscores the importance of developing targeted prevention strategies and adapting patient counseling to alleviate the impact of diverticula. Future research should focus on clarifying the underlying pathophysiological mechanisms connecting these conditions and evaluating the effectiveness of tailored interventions in reducing the burden of this common disorder.

## Figures and Tables

**Table 1:** Baseline characteristics of patients with low–moderate risk, high risk, and very high risk, calculated with SCORE2. Most continuous variables were non-normally distributed. Continuous data are given as median ± interquartile range (IQR) and compared using the Mann–Whitney U-test or mean ± standard deviation (SD) and compared using Student’s *t*-test accordingly. Categorical data are given as numbers (percentages) and compared using the chi-square test. All tests were two-sided, and a *p*-value of <0.05 was considered statistically significant.

	Total	Low–Moderate Risk	High Risk	Very High Risk	*p*-Value
	N = 3935	N = 1512	N = 1651	N = 772	
AGE	56 (51–61)	53 (50–56)	57 (51–62)	63 (58–67)	<0.001
<50	17% (685)	21% (321)	19% (318)	6% (46)	
50–69	83% (3250)	79% (1191)	81% (1333)	94% (726)	
SEX					<0.001
Female	45% (1756)	71% (1080)	32% (525)	20% (151)	
Male	55% (2179)	29% (432)	68% (1126)	80% (621)	
BMI	26 (24–30)	25 (22–28)	27 (25–30)	28 (26–32)	<0.001
BMI ≥ 30	23% (913)	14% (217)	24% (403)	38% (293)	
BMI 25 to <29	41% (1616)	34% (512)	47% (774)	43% (330)	
BMI < 25	36% (1406)	52% (783)	29% (474)	19% (149)	
Hypertension	53% (2088)	31% (464)	59% (980)	83% (644)	<0.001
RR ≥ 140 or ≥90 mmHg	43% (1689)	23% (355)	47% (783)	71% (551)	
RR intermediate	53% (2070)	69% (1045)	49% (816)	27% (209)	
RR < 120/80 mmHg	4% (176)	7% (112)	3% (52)	2% (12)	
Kreatinin	0.9 (0.8–1.0)	0.8 (0.8–0.9)	0.9 (0.8–1.0)	0.9 (0.8–1.0)	<0.001
HGB	14.7 (13.9–15.5)	14.2 (13.5–14.9)	15.0 (14.3–15.7)	15.2 (14.3–15.9)	<0.001
MCV	87 (85–90)	87 (84–90)	87 (85–89)	88 (85–90)	0.003
Thrombo	232 (201–270)	243 (211–278)	229 (198–265)	219 (190–258)	<0.001
Leuko	5.8 (4.9–7.0)	5.5 (4.7–6.5)	5.8 (5.0–7.1)	6.4 (5.3–7.6)	<0.001
CRP	0.2 (0.1–0.3)	0.1 (0.1–0.3)	0.2 (0.1–0.3)	0.2 (0.1–0.5)	<0.001
BSG	5 (2–9)	5 (2–8)	5 (2–8)	6 (3–10)	<0.001
Cholesterin	221 (193–249)	218 (192–244)	224 (197–253)	218 (186–254)	<0.001
Cholesterol ≥ 240 mg/dL	33% (1290)	30% (451)	36% (587)	33% (252)	
Cholesterol 200 to 239 mg/dL or treated	46% (1793)	43% (647)	46% (761)	50% (385)	
Cholesterol < 200 or untreated	22% (852)	27% (414)	18% (303)	17% (135)	
LDL	141 (117–168)	136 (113–159)	147 (123–173)	143 (114–173)	<0.001
HDL	55 (46–66)	63 (53–74)	52 (45–62)	47 (40–56)	<0.001
Metabolic syndrome	77% (3013)	62% (937)	82% (1360)	93% (716)	<0.001
Smoking status					<0.001
Never smoker	31% (1222)	39% (584)	28% (468)	22% (170)	
Ex-smoker	42% (1636)	47% (705)	41% (673)	34% (258)	
Active smoker	27% (1064)	14% (217)	31% (505)	44% (342)	

**Table 2:** (**a**) Endoscopic characteristics of patients with low–moderate, high, and very high cardiovascular risk according to SCORE2. Most continuous variables were non-normally distributed. Continuous data are given as a median ± interquartile range (IQR) and compared using the Mann–Whitney U-test or mean ± standard deviation (SD) and compared using Student’s *t*-test accordingly. Categorical data are given as numbers (percentages) and compared using the chi-square test. All tests were two-sided, and a *p*-value of <0.05 was considered statistically significant. (**b**) ORs of the endoscopic characteristics of patients with low–moderate, high, and very high cardiovascular risk according to SCORE2.

(a)	Low–Moderate Risk	High Risk	Very High Risk	*p*-Value
	N = 1512	N = 1651	N = 772	
Diverticulosis	24% (357)	38% (630)	44% (339)	<0.001
No diverticulosis	76% (1155)	62% (1021)	56% (433)	
Left-sided diverticulosis	17% (253)	26% (427)	25% (193)	
Right-sided diverticulosis	3% (40)	4% (71)	7% (53)	
Pandiverticulosis	4% (64)	8% (132)	12% (93)	
**(b)**	**Low–Moderate Risk**	**High Risk**	**Very High Risk**	
		OR	OR	
Diverticulosis	ref	1.99	2.53	
No diverticulosis	ref	0.50	0.40	
Left-sided diverticulosis	ref	1.74	1.66	
Right-sided diverticulosis	ref	1.66	2.71	
Pandiverticulosis	ref	1.97	3.10	

**Table 3:** Comparison of the high-risk group and the very-high-risk group with the low/moderate-risk group as the reference in the unadjusted Model-1 and the Model-2 adjusted for metabolic syndrome.

	Low/Moderate Risk	High Risk	Very High Risk	R^2^
Model-1	Ref.	OR 2.00; 95%CI 1.71–2.33; *p* < 0.001	OR 2.53; 95%CI 2.10–3.05; *p* < 0.001	0.0243
Model-2	Ref.	aOR 1.86; 95%CI 1.59–2.18; *p* < 0.001	aOR 2.27; 95%CI 1.88–2.75; *p* < 0.001	0.0280

## Data Availability

The data presented in this study are available on request from the corresponding author due to ethical considerations and privacy concerns.
